# Surgically Treated Cervical Cancer in Manitoba: A Retrospective Study of the Impact of Geography on Care

**DOI:** 10.3390/curroncol33020070

**Published:** 2026-01-25

**Authors:** Nora-Beth Mercier, Yuliia Khudina, Lesley Roberts, Allison Feely, Oliver Bucher, Pascal Lambert, Alon D. Altman

**Affiliations:** 1 Division of Gynecologic Oncology, Winnipeg Women’s Hospital, University of Manitoba, Winnipeg, MB R3E 0L8, Canada; 2 Department of Epidemiology and Cancer Registry, CancerCare Manitoba, Winnipeg, MB R3E 0V9, Canada; 3 Paul Albrechtsen Research Institute, CancerCare Manitoba, Winnipeg, MB R3E 0V9, Canada

**Keywords:** cervical cancer, surgical treatment, geographic impact, overall survival, recurrence-free survival

## Abstract

Background/Objectives: Cervical cancer outcomes based on geographic location of residence reveal inconsistent patterns, and most of the evidence is from the United States. This retrospective study aimed to investigate whether there existed a difference in overall survival (OS) and recurrence-free survival (RFS) between individuals living within a Canadian city with a tertiary care centre versus those living remotely within a large catchment area (up to >1000 km travel distance), including a sizeable rural component. Methods: Surgically treated cervical cancer patients from 2000 to 2016 were included. Patients were treated with either radical hysterectomy, trachelectomy, or simple hysterectomy. Adjuvant treatment was provided depending on surgical pathology. OS and RFS were estimated using Kaplan–Meier curves and cumulative incidence curves. Results: Two hundred and eighty-two patients with surgically treated cervical cancer were included: 185 patients living within urban city limits and 97 patients living rurally. There were no significant baseline differences between groups. No significant difference in OS or RFS was found, even after adjusting for death as a competing risk for RFS. The median time to surgery for residents living within versus outside the city was 84 vs. 66 days, respectively, although this difference was not statistically significant (*p* = 0.3179). Conclusions: This is the first Canadian study to examine an association between survival and distance to care for cervical cancer.

## Simple Summary

Despite advances in cervical cancer screening, rates of cervical cancer in Canada have risen. Traditionally, distance to care has been associated with worse disease outcomes. Yet, outcomes based on the geographic location of the patients’ residence when it comes to cervical cancer, mainly out of the United States, have revealed inconsistent patterns. In patients with primary surgically treated cervical cancer, the study aimed to investigate if a difference in overall survival or recurrence-free survival existed between individuals living within a city containing a tertiary care centre compared to those living outside city limits. Results suggested no significant geographic disparity among patients treated at one centre. Further studies are needed to strengthen this inference, given this is the first Canadian study to examine the association between survival and distance to care for cervical cancer. Additional work should also account for further demographic characteristics such as race, ethnicity, education, and socioeconomic status.

## 1. Introduction

Cervical cancer is the fourth most prevalent cancer in women aged 15–44 [1], with roughly 1600 individuals being diagnosed and 400 individuals having died of cervical cancer in 2024 alone [2]. This makes it the third most deadly cancer in Canadian women 15–44 years of age [1]. Despite being largely preventable, rates of cervical cancer have been increasing in Canada in recent years [3]. While the prevalence of cervical cancer varies across the country, Manitoba has the highest rate across the ten provinces (10.1 per 100,000) [3]. The second highest was Alberta at 8.2 per 100,000, with Nova Scotia citing the lowest rates of all Canadian provinces at 6.4 per 100,000 [3]. Incidence rates across the country have continued to be higher among lower-income communities, rural locations, and within Indigenous communities, which may factor into Manitoba’s higher numbers. Within Manitoba, all the gynecologic oncologists work out of CancerCare Manitoba (CCMB), which is based in Winnipeg. This is also where most of the care is administered/coordinated for Manitoba, as well as for parts of Nunavut and parts of North-Western Ontario. Between 2000 and 2016, eight hundred and fifty-two cervical cancers were diagnosed in Manitoba. Over half (57.4%) were treated with primary surgery with or without adjuvant treatment. Twenty-nine and half percent had concurrent chemoradiation therapy (CCRT), 12.9% had radiation therapy alone, and 0.2% had chemo alone.

The impact of distance to care for cancer patients has been a well-established research topic for years. Papers have compared screening rates, clinical stages at diagnosis, and overall survival outcomes for rural and urban populations. A 2019 systematic review from Australia suggested worse survival for cancer patients living in rural areas in that country. The evidence was most consistent for colorectal, lung, and prostate cancers [4]. Within gynecologic oncology, geographic disparities have also been examined, with much of the early research focused on ovarian cancer. Thus far, the evidence suggests improved outcomes with access to expert gynecologic oncology care, which is most often located in larger urban centres [5].

When it comes to cervical cancer and place of residence, previous results in the literature have been inconsistent. One of the initial studies performed by Gunderson et al. in 2013 suggested that there was actually no difference in recurrence-free survival (RFS) or overall survival (OS) for patients when comparing distances travelled for their cancer care [6]. While many subsequent papers have shown similar outcomes, others, on the other hand, have suggested the exact opposite, including higher stage, delays to treatment, and worse mortality rates for those from rural areas diagnosed with cervical cancer [5,7,8,9,10,11,12,13]. Unfortunately for Canadians, all of these studies arise from outside of the country, making generalizability difficult. To our knowledge, this would be the first study from a Canadian population to investigate this possible association.

This study aims to investigate whether there exists a difference in overall and recurrence-free survival between individuals in Manitoba with primary surgical treatment of cervical cancer living outside of Winnipeg compared to those residing within city limits. Our secondary objective is to determine if there is a difference in time to surgery for those outside the city, and, if so, whether it is related to outcomes.

## 2. Materials and Methods

This is a retrospective cohort study of patients treated with primary surgery for cervical cancer between January 2000 and December 2016 from the Manitoba Cancer Registry. Individuals were included if they were older than 18 years of age and if they had been treated with primary surgery for cervical cancer, including radical or simple hysterectomy, or radical or simple trachelectomy. Individuals were excluded if they had received any neoadjuvant chemotherapy or radiotherapy and if no to minimal data could be recovered. Ethics approval was obtained from the University of Manitoba Health Research Ethics Board (REB #HS23724).

The Manitoba Cancer Registry was used to identify cervical cancer cases diagnosed between 2000 and 2016 and treated with surgery. An individual’s date and postal code at diagnosis, tumour site, morphology, treatment type, and treatment date were identified. Data was collected within a REDCap database housed within a secure research environment. Both electronic and paper medical records, where applicable, were obtained and reviewed, and data was collected both in accordance with the 4C initiative and to meet the objectives of this paper. Extracted data included age at diagnosis, history of smoking, American Society of Anesthesiology (ASA) class, height, weight, BMI, 2018 FIGO (International Federation of Gynecology and Obstetrics) stage, preoperative imaging, date of biopsy, date of surgery, type of surgery, history of conversion of surgery, cervical approach/type (i.e., radical vs. simple hysterectomy), lymph node assessment, use of intrauterine manipulator, histology, intraoperative complications, postoperative complications, length of hospital stay (LOS), readmission and ER visits, specimen integrity, parametrial and vaginal involvement, tumour size (clinical, radiological, and pathological), stromal invasion, lymphovascular space invasion (LVSI), surgical margin and distance from the margin, lymph node metastases, adjuvant treatment, recurrence date and location, date of last follow-up, date of death, cause of death, primary treatment site/centre, and lastly, variables reflective of enhanced recovery after surgery (ERAS) protocols including time to Foley catheter removal, time to mobilization, postoperative diet, venous thromboembolism (VTE) prophylaxis, pain regimes, time to fluid discontinuation, and postoperative ileus prevention.

Winnipeg residents were defined as those having a postal code at cancer diagnosis within the Winnipeg Regional Health Authority (WRHA), excluding any residents of Churchill, Manitoba (a northern community served by the WRHA). The classification of postal codes was defined using the standard definitions employed by Manitoba Health and the Manitoba Centre for Health Policy. Surgical approach was decided by the patients’ primary surgeon. A combined vaginal laparoscopic approach (LAVH) consisted of completion of the hysterectomy by laparoscopy up to the colpotomy, with the colpotomy and cuff closure then being performed vaginally. Any adjuvant therapy required postoperatively was determined by a gynecologic oncologist affiliated with CancerCare Manitoba. All patients received a uniform minimum follow-up period of 5 years before being discharged from cancer care back to primary care for annual examinations (unless lost to follow-up). This did not vary based on the year of diagnosis. Recurrence was established based on the presence of disease on either imaging or biopsy. Overall survival (OS) was defined as time from surgery to death from any cause. Recurrence-free survival (RFS) was also calculated from the date of surgery but was defined and assessed in two ways: (1) using a definition of recurrence or death, whichever occurred first, and (2) using recurrence as the sole definition and using death as a competing risk.

Descriptive statistics were used to summarize demographic, operative, pathologic, treatment, and outcome variables. For categorical variables, differences were assessed using either the Chi-square test or Fisher’s exact test, the latter being used when the expected frequencies required it. To compare the median time to surgery between those within Winnipeg versus those outside the city limits, the Wilcoxon test was used. Overall survival, RFS (using definition (1) above), and time to surgery were compared with the log-rank test using Kaplan–Meier curves. Recurrence-free survival using death as a competing risk was compared with Gray’s test using a cumulative incidence curve. Given the small number of events in the cohort, a multivariable Cox proportional hazards model for overall survival was not performed. To avoid potential misleading conclusions from multiple comparisons, the determination of statistical significance was performed in a post hoc manner to limit the false discovery rate at 5% using the method described by Benjamini and Hochberg [14]. SAS 9.4, Stata 18, and R 4.4.0 were the statistical software used to conduct the analysis. The STROBE checklist was used to aid in creation of this manuscript [15].

## 3. Results

**Chart 1:**
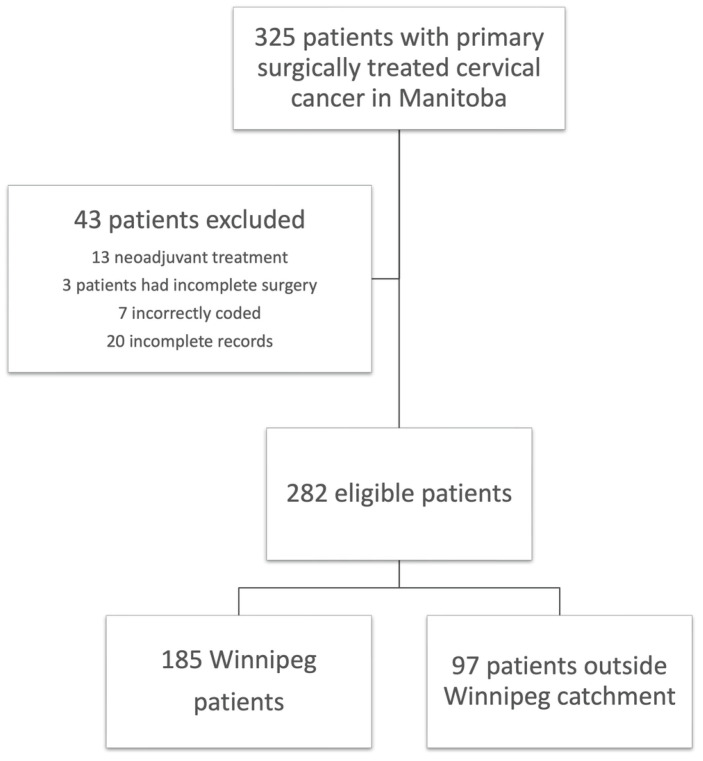
Study flow diagram.

Between January 2000 and December 2016, 325 patients who had surgically treated cervical cancer in Manitoba were identified. Of those, 13 patients were excluded because they had neoadjuvant treatment prior to surgery, 3 patients did not have a completed hysterectomy or trachelectomy, 7 patients were incorrectly coded and did not have surgical management, and 20 patients were excluded due to incomplete data records. The final count was 282 eligible patients within the database: 185 patients were Winnipeg residents, and 97 patients resided elsewhere within the province’s catchment area (Chart 1). Table 1 contains all of the cohort’s characteristics stratified by residence status.

Overall, 85.1% of patients had FIGO 2018 Stage I disease. Squamous cell carcinoma (SCC) was the most common histology at 55%, followed by adenocarcinoma at 35.8%, adenosquamous carcinoma at 3.9%, and other pathologies at 5.3%. The majority of patients (61.3%) were between the ages of 30 and 49 years at the time of diagnosis. Only 5.3% of the cohort were 70 years of age or older. Sixty-one percent of patients had a radical hysterectomy, while 36.9% underwent a simple hysterectomy, and only 2.1% had a radical trachelectomy. The surgical approach in the vast majority of cases was open (81.9%), while 7.8% cases were laparoscopic. Of the two surgical sites in Winnipeg, 61.7% of patients received their surgical care at the Health Sciences Center, and 29.4% were treated at St. Boniface General Hospital. The remainder of the cohort had their procedure completed elsewhere. The intra-operative complication rate was 13.8%, and postoperative complications were documented in 36.5% of patients, although a substantial amount either had missing or no documentation of this (37.6%). The median length of stay was 4 days. Emergency room visits within 30 days post-surgery occurred in 13.5% of the cohort, while only 7.8% required readmission within this timeframe. Given the early stage at diagnosis for the majority of patients, 63.8% did not receive any adjuvant treatment. For those who did, 14.2% were treated with radiation, 1.1% with chemotherapy, and 13.5% with CCRT. Recurrence was documented in 36 individuals (12.8%) and death in 40 patients (14.2%). When stratifying the cohort by place of residence and adjusting to limit the false discovery rate at 5%, there were no significant differences between in the groups in any of the above-mentioned characteristics (Table 1).

**Figure 1:**
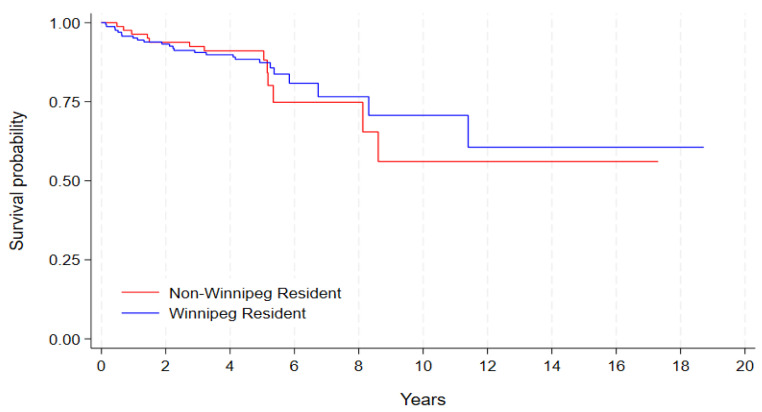
Kaplan–Meier curve for overall survival by Winnipeg residence.

**Figure 2:**
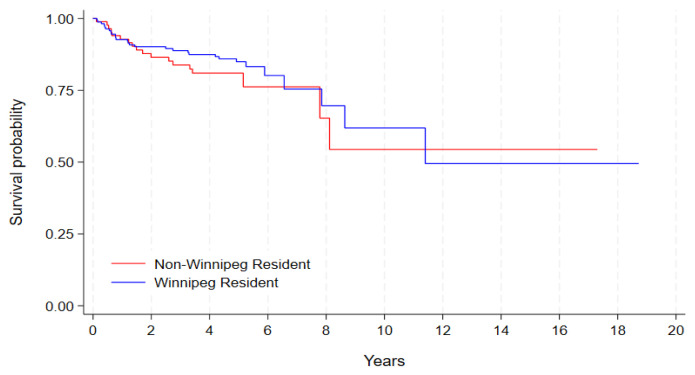
Kaplan–Meier curve for recurrence-free survival by Winnipeg residence.

**Figure 3:**
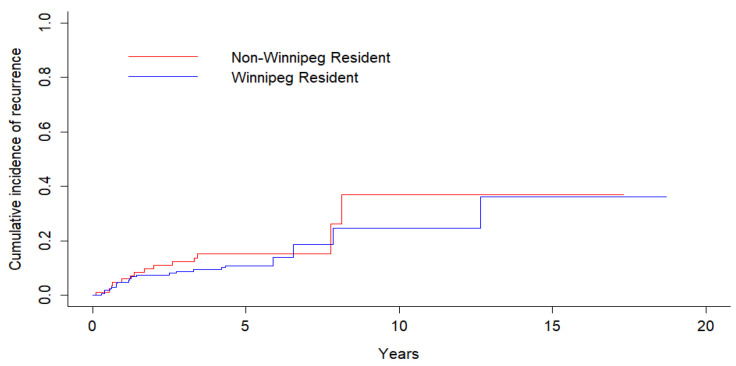
Cumulative incidence curve for recurrence by Winnipeg residence.

Overall survival and RFS between the two groups were not significantly different (Figure 1 and Figure 2). Twenty-four patients were removed from the analysis: 23 had missing follow-up dates and were lost to follow-up, while one patient had a chronological inconsistency between surgery and follow-up dates that could not be reconciled (i.e., they had a negative survival time). Overall survival was 76.8% among Winnipeg residents and 77.3% for those living outside the city limits, with corresponding survival functions of 0.71 (95% CI 0.52–0.83) and 0.56 (95% CI 0.28–0.77) at 10 years, respectively (log-rank *p* = 0.8225; Figure 1). Recurrence occurred in 14 (14.4%) non-Winnipeg residents and 22 (11.9%) residents living within Winnipeg. Cumulative incidence of recurrence between the groups with death as a competing risk factor was not statistically different (k-sample *p* = 0.3483; Figure 3), and neither was RFS (log-rank *p* = 0.3968; Figure 2). Since event rates were small, multivariable analysis was not performed due to the lack of power and stability in the parameter estimates.

**Figure 4:**
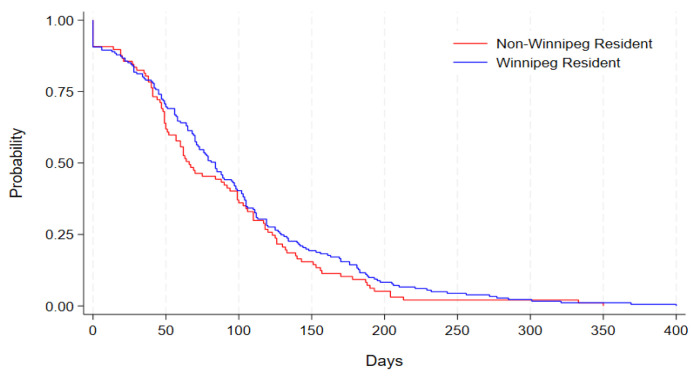
Kaplan–Meier curve for time to surgery by Winnipeg residence.

Time to surgery was calculated from the date of diagnosis (in days). Four outliers from the data were removed from the analysis, given these individuals had surgery beyond 900 days after their initial diagnosis. Their inclusion would have made interpretation of the results more difficult. Two of the four patients had multiple cone biopsies while being followed in colposcopy (one with close margins and the other with a pathology review that eventually showed invasion). A third patient wanted more children initially and had a cone with 2.25 mm depth of invasion with negative margins. The details of delay in diagnosis to surgery for the final patient are unclear, but diagnosis was in 2007 with surgery in 2010. The median time to surgery for residents living outside city was 66 days (IQR 41–123 days) versus 84 days (IQR 45–129 days) for those in the city, although this was not statistically significant using both the Wilcoxon and log-rank tests (*p* = 0.3179 and *p* = 0.2303, respectively; Figure 4).

## 4. Discussion

The impact of geography on medical care, including cancer care, has been an area of research for decades and represents a well-known social determinant of health. In gynecologic oncology, much of the early work focused on ovarian cancer, and improved outcomes were associated with timely access to expert, high-volume gynecologic oncologic care [5,13,16]. Naturally, researchers hypothesized similar results when it came to cervical cancer; however, the evidence has been somewhat contradictory.

Yu and colleagues released a large study (n = 59,432) that found overall cervical cancer incidence and mortality rates were higher amongst rural/non-metropolitan patients within the United States. They also noted that the difference in incidence rates varied in both urban and rural settings depending on race [11]. This finding was also found in a recent American study by Amboree et al., which found a recent trend of increasing cervical cancer incidence in rural US counties, which the authors felt was likely related to lower screening rates and less suspected access to therapeutics such as vaccination [17]. Furthermore, Barrington et al. performed a retrospective study of 390 cervical cancer patients looking at distance to a single comprehensive cancer centre in Alabama and found poorer OS and PFS in patients residing ≥ 100 miles from the tertiary centre [13]. With those studies in mind, there are several other papers, including our study, that counter these conclusions. The largest study on this topic, performed by Steele et. al, was a multivariable analysis of over 50,000 cervical patients from the National Cancer Database in the U.S. This study showed that greater distance to care was associated with a stepwise risk reduction in diagnosing advanced disease. Unfortunately, they did not investigate OS or PFS outcomes [8]. Meanwhile, a paper by Gunderson and colleagues was one of the first to find no difference in four-year OS and PFS, even among patients travelling greater than 50 miles for care [6]. The same conclusion was drawn in a study from Virginia looking specifically at differences in outcomes for locally advanced cervical cancer and travel distance to concurrent chemotherapy and radiation treatments [5]. Over the past five years, more work out of the United States continued to be published with the same conclusions, even suggesting improved outcomes for rural/remote patients [7,9,10].

Although not statistically significant, our median time to surgery for non-Winnipeg residents was shorter at 66 days versus 84 days for Winnipeg residents, further supporting the existing literature in favour of no difference (of note, the IQR and ranges were otherwise quite similar). The exact reason for this trend remains unclear and should only be seen as hypothesis generating. One possible explanation could be that rural patients tend to receive supports such as nurse navigators, employed by CancerCare Manitoba to assist with transportation services and prioritize this to reduce travel-related delays. Other potential factors such as disparities in socioeconomic status, education, childcare, and other systemic or logistical barriers could also be affecting timely care. Interestingly, a recent large retrospective cohort study in the United States also found a paradoxical effect of rural counties accessing care earlier (albeit only by an average of 8.8 days) than urban residence [18]. This was also previously found in a cohort by Spees and colleagues, who found that greater distance appeared to be unfavourable among urban residents yet seemed protective among rural residents—they hypothesized that a higher proportion of rural residents owning personal means of transportation was likely the main driver for this effect [10]. Further studies could investigate the potential role of socioeconomic status, race, ethnicity, education, and access to technology in contributing to these differences. Additionally, part of the differences in results could stem from the amount of heterogeneity in the different cohorts seen across the studies previously published. If, for example, rural areas have more advanced disease at the time of diagnosis, then worse outcomes would be expected for rural patients if rural and urban patients are all included. For studies that limited the cohorts to a treatment group, this heterogeneity between urban and rural patients can be diminished due to the treatment inclusion criteria. This makes it more difficult to detect significance, which may have been the case in our study, which only looked at surgically treated cervical cancer.

There are several limitations to this study. The first and most important is its retrospective nature, which brings along significant risks of confounding and biases but is difficult to avoid given the limited feasibility to perform a prospective study on this topic. Furthermore, our small sample significantly reduced statistical power, and the small event size did not permit the use of reliable multivariable analyses to help account for potential confounders. With regard to the minimum detectable difference in our study, an event-based (Freedman) approximation using our data implies we had an ~80% power to detect a hazard ratio (HR) of 2.60 (or 0.38) at an alpha of 0.05. When accounting for multiplicity using the most conservative threshold of limiting the FDR to <5% (i.e., the Bonferroni correction, or q/m = 0.003125), the detectable effect increases to an HR of 3.66 (or 0.27). Additionally, other likely important variables, such as key demographic characteristics including race, ethnicity, socioeconomic status, and education, were not captured. Another shortcoming is that it is a single-centre review within one province, thus limiting applicability to the entire Canadian population. Finally, expanding our study to look at numerical distance to care as opposed to categorically grouping individuals as Winnipeg residents or non-residents would have also been propitious. By not doing this, we may have not been able to accurately capture the difference in outcomes for patients living thousands of kilometers from Winnipeg versus those residing only slightly beyond city limits. Furthermore, patients’ residence may have changed from the initial postal code recorded at diagnosis, or they may have temporarily stayed with friends or relatives during treatment, leading to possible misclassification. The study duration spanning more than 15 years also potentially introduces temporal heterogeneity into the analysis given potential advances in surgical techniques, perioperative management, and referral pathways that may have occurred. Lastly, the exclusions mentioned above coupled with missing perioperative data further introduce biases into the study, potentially influencing survival estimates and limiting assessment of postoperative outcomes.

Given that the majority of the literature found comes from the United States, our work provides results from a Canadian perspective and within a public health care system. Another strength of this study is the follow-up time: with the last patients included undergoing surgery in 2016 and data extraction continuing until 2023, this allowed for a prolonged period to capture recurrence and all-cause mortality. Although definitive conclusions cannot yet be drawn, this study contributes to the existing literature and may help stimulate further research within Canada. CancerCare Manitoba in Winnipeg is unique given it is the only tertiary centre for the province and has the largest catchment area in the country, with all care delivered centrally. A further advantage is the quality of the Manitoba Cancer Registry, which regularly obtains gold certification by the North American Association of Central Cancer Registries [19]. The conflicting results in this area continue to demonstrate the complexities in understanding how geographic impact and distance contribute to cervical cancer outcomes. There is speculation that even just coordination of care at a tertiary cancer centre may lead to improved outcomes and mitigate the effects of distance (as is the case in Winnipeg, with a centralized system at one tertiary centre). [5,20]. It also remains to be seen whether the introduction of advanced telecommunications and technology may influence the patient experience.

## 5. Conclusions

To our knowledge, this is the first Canadian study to examine an association between survival and distance to care for cervical cancer. Although this study suggests no significant geographic disparity among cervical cancer patients treated at one centre within Manitoba, further studies are needed to strengthen this inference, especially as it relates to a Canadian context. Lastly, additional work should also account for other important demographic characteristics such as race, ethnicity, education, socioeconomic status, and numerical distance to primary care site.

## Figures and Tables

**Table 1:** Cohort characteristics stratified by Winnipeg and non-Winnipeg residents.

	Full Cohort		Winnipeg Resident				
			No		Yes		
	N	Col %	N	Col %	N	Col %	* p*-Value
Total	282	100.0	97	100.0	185	100.0	
Year of birth							
1920–1939	17	6.0	s	s	s	s	0.2455
1940–1949	29	10.3	s	s	s	s	
1950–1959	59	20.9	20	20.6	39	21.1	
1960–1969	99	35.1	29	29.9	70	37.8	
1970–1979	59	20.9	23	23.7	36	19.5	
1980–2001	19	6.7	11	11.3	8	4.3	
Year of diagnosis							
2000–2004	85	30.1	34	35.1	51	27.6	0.0133
2005–2009	85	30.1	20	20.6	65	35.1	
2010–2014	80	28.4	26	26.8	54	29.2	
2015–2016	32	11.3	17	17.5	15	8.1	
Histology							
Adenocarcinoma	101	35.8	36	37.1	65	35.1	0.4992
Adenosquamous carcinoma	11	3.9	s	s	s	s	
Squamous cell carcinoma	155	55.0	50	51.5	105	56.8	
Other	15	5.3	s	s	s	s	
Age at diagnosis							
20–29	19	6.7	8	8.2	11	5.9	0.4509
30–39	77	27.3	31	32.0	46	24.9	
40–49	96	34.0	30	30.9	66	35.7	
50–59	46	16.3	16	16.5	30	16.2	
60–69	29	10.3	6	6.2	23	12.4	
70+	15	5.3	6	6.2	9	4.9	
Disease Stage							
Stage I	240	85.1	85	87.6	155	83.8	0.7266
Stage II	s	s	s	s	s	s	
Stage III/IV	25	8.9	6	6.2	19	10.3	
Missing	s	s	s	s	s	s	
Year of surgery							
2000–2004	78	27.7	32	33.0	46	24.9	0.0078
2005–2009	84	29.8	21	21.6	63	34.1	
2010–2014	85	30.1	25	25.8	60	32.4	
2015–2017	35	12.4	19	19.6	16	8.6	
Cervical surgery type							
Radical hysterectomy	172	61.0	60	61.9	112	60.5	0.9655
Radical trachelectomy	6	2.1	s	s	s	s	
Simple hysterectomy	104	36.9	s	s	s	s	
Surgical approach							
Combined vaginal/laparoscopic	s	s	s	s	s	s	0.0615
Laparoscopic	22	7.8	7	7.2	15	8.1	
Open	231	81.9	86	88.7	145	78.4	
Vaginal	10	3.5	0	0.0	10	5.4	
Missing	s	s	s	s	s	s	
Primary surgical site							
Brandon	s	s	s	s	s	s	0.0094
Health Sciences Center—Women’s Hospital	174	61.7	58	59.8	116	62.7	
St. Boniface General Hospital	83	29.4	25	25.8	58	31.4	
Other	18	6.4	9	9.3	9	4.9	
Missing	s	s	s	s	s	s	
Recurrence							
Yes	36	12.8	14	14.4	22	11.9	0.8161
No	209	74.1	70	72.2	139	75.1	
Missing	37	13.1	13	13.4	24	13.0	
Vital status							
Alive	217	77.0	75	77.3	142	76.8	0.9643
Dead	40	14.2	14	14.4	26	14.1	
Missing	25	8.9	8	8.2	17	9.2	

None of these *p* values were significant when stratifying the cohort by place of residence and adjusting to limit the false discovery rate at 5% (i.e., *p* < 0.003125 would be necessary to reach statistical significance). ‘s’ signifies counts between 1 and 5 that were suppressed as per local Institutional Review Board/ethics requirements.

## Data Availability

Data is stored on an institutional secure REDCap database, complying with local REB. Raw data can be made available on reasonable request.
